# Bidirectional association between depression and diabetic nephropathy by meta-analysis

**DOI:** 10.1371/journal.pone.0278489

**Published:** 2022-12-20

**Authors:** Tingting Fang, Qiuling Zhang, Zhiguo Wang, Jun-Ping Liu

**Affiliations:** 1 Institute of Ageing Research, Hangzhou Normal University, School of Basic Medicine, Hangzhou, Zhejiang Province, China; 2 School of Public Health, Hangzhou Normal University, Hangzhou, Zhejiang Province, China; 3 Department of Endocrinology, The Affiliated Hospital of Hangzhou Normal University, Hangzhou, Zhejiang Province, China; 4 Monash University Department of Immunology and Pathology, Central Clinical School, Faculty of Medicine, Prahran, Victoria, Australia; 5 Hudson Institute of Medical Research, Clayton, Victoria, Australia; The University of the West Indies, JAMAICA

## Abstract

**Background:**

Studies suggested that the association between depression and diabetic nephropathy may be bi-directional, but this hypothesis remains investigating. In this meta-analysis, the bi-directional relationship between depression and diabetic nephropathy was investigated.

**Methods:**

A search for the publications on depression and diabetic nephropathy in the databases of PubMed, Web of science, and Embase from the earliest available to August 2022 was conducted. Two sets of pooled risk estimates were calculated using random effects models: diabetic nephropathy predicting depression and depression predicting diabetic nephropathy. Cross-sectional studies were assessed using Agency for Healthcare Research and Quality (AHRQ), cohort and case-control studies were assessed using Newcastle-Ottawa Scale (NOS).

**Result:**

Of the 974,121 patients in 30 clinical studies, 24 studies met eligibility for diabetic nephropathy predicting onset of depression, representing 28,438 incident cases. The other 6 studies met criteria for depression predicting onset of diabetic nephropathy, representing 945,683 incident cases. The pooled odds ratio (OR) of diabetic nephropathy predicting depression was 1.46 (95% CI 1.27–1.67). The OR of depression predicting diabetic nephropathy was 1.22 (95% CI 1.13–1.31).

**Conclusion:**

This meta-analysis shows that the relationship between depression and diabetic nephropathy may be bidirectional. Diabetic nephropathy may be a predictor of depression, and depression may also be an indicator of diabetic nephropathy. The mechanisms underlying the bidirectional relationship need to be further investigated and interventions of the comorbidity of depression and diabetic nephropathy need be studied in clinical practice.

## Introduction

The global disease diabetes imposes threat to many aspects of public health. From 2005 to 2015, the cases of diabetes increased from 333 million to 435 million, predicting 439 million diabetics worldwide by 2030 [1, 2]. Of a variety of microvascular and macrovascular complications, including blindness, kidney disease, and lower limb amputation, diabetic nephropathy (DN) is the most common microvascular complication of diabetes. In the United States, the number of patients with end-stage renal failure (ESRD) due to DN increased from 40,000 to 50,000 between 2000 and 2014 [3]. Compared with the developed countries, greater burden of chronic kidney disease due to diabetes was in developing countries [4]. In the past decades, the occurrence of DN in China increased dramatically, with 24.3 million patients suffering from diabetic kidney disease in 2016 [5]. Studies showed that 95% of diabetics developed kidney injury after 10 years, 35% progressed to end-stage kidney disease after 5 years, and 18% died of kidney failure after 20 years [6]. In addition to the organic complications, diabetes underpins severe mental disorders such as depression and anxiety, the prevalence of depression was doubled in type 2 diabetics compared with subjects without diabetes [7].

Depression is a most common mental disorders, recently dramatically increased worldwide [1]. Depression is associated with unhealthy behaviors such as smoking, lack of exercise, and calorie intake, reducing life quality and diabetes self-care ability [8–10]. Depression-related pathophysiological mechanisms include dysregulation of hypothalamic-pituitary-adrenal-immune (HPAI) axis and activation of pro-inflammatory cytokines, potentially resulting in insulin resistance and increased risk of diabetes [9, 11].

The present study was undertaken to systematically review the reported data on DN and depression by performing meta-analysis. We found that DN and depression coexist as comorbidity developing shared risk of intimately influencing each other [12].

### Research question

- Whether diabetic nephropathy is a risk factor of depression?
- Whether depression is a risk factor of diabetic nephropathy?
- Is there a bidirectional relationship between diabetic nephropathy and depression?

## Methods

We followed the Preferred Reporting Items for Systematic Reviews and Meta-Analysis (PRISMA) checklist (S1 Checklist) [13], and registered in the international prospective register of systematic reviews (PROSPERO) under the registration number CRD42022357342.

### Inclusion and exclusion criteria

Included in the analysis were those that met the following criteria: (1) the study design was a cohort, case-control, cross-sectional study; (2) contained DN and depression (depressive disorders and symptoms, not only the syndrome of major depression); (3) reported odds ratios (ORs), relative risks (RRs) or hazard ratios (HRs) with corresponding 95% confidence intervals (CI); and (4) published in English. Exclusion criteria: (1) duplicate literature; (2) reviews, conferences abstracts, case reports, animal experiments; and (3) randomized controlled trial study (RCTs).

### Search strategy

**Fig 1:**
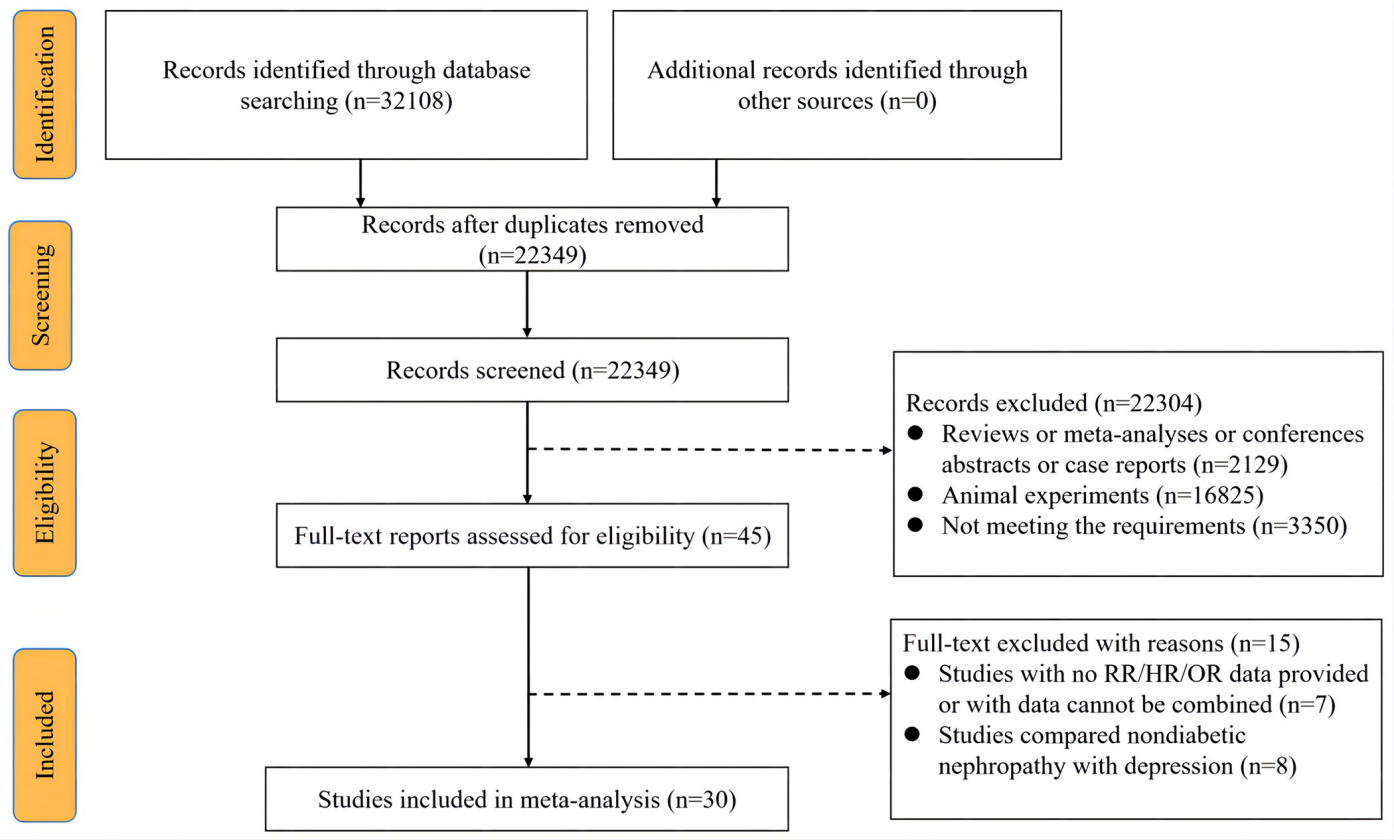
Systematic review flow chart.

PubMed, Embase and Web of Science databases were searched for related studies the earliest available online to 31 August 2022. A text search with the following keywords singly or in combination was conducted: ‘Diabetic Nephropathy’, ‘Diabetic Kidney Disease’, ‘Chronic Kidney Disease’, ‘Nephropathy’, ‘Nephrosis’, ‘DN’, ‘DKD’, ‘Diabetes Mellitus’, ‘Diabetes Mellitus, Type 2’, ‘Diabetes Mellitus, Type 1’, ‘Depression’, ‘Emotional Depression’, ‘Tristimania’, ‘Depressive Symptom’. The detailed search strategy was shown in Fig 1. Additionally, we also searched and retrieved the references of all included studies [14].

### Data extraction

Data extraction was conducted with the information including the following: (1) name of the first author, (2) publication year, (3) country, (4) study design, (5) sample size, (6) age, (7) method of depression assessment, (8) diagnostic methods of DN, (9) adjusted odds ratios (ORs), relative risks (RRs) or hazard ratio (HRs) with 95% CI. The DN assessment criteria was by (1) albuminuria, (2) estimate glomerular filtration rate (eGFR), (3) urinary albumin-to-creatinine ratio (UACR), and (4) urinary albumin excretion rate (UAER). The depression assessment criteria was by (1) diagnostic interview, (2) self-reported questionnaire, and (3) physician diagnosis [14]. In selected studies, when DN was divided into different grades according to the degree of renal impairment, the OR/RR/HR values of the most severe group were selected for summary analysis. In order to control the influence of confounding factors, when there were multiple adjusted OR/HR/RR values, the most adjusted value was selected for summary analysis [14].

### Quality assessment

Quality control of cross‐sectional studies was evaluated using the assessment involving 11 items recommended by the Agency for Healthcare Research and Quality (AHRQ). The total score ranged from 0 to 11, with a score of 8 or higher considered high quality (S1 Table in S1 File) [15]. For cohort and case-control studies, we utilized the Newcastle-Ottawa Scale (NOS), where total scores range from 0 to 9 with a score of 7 or higher regarded as high quality (S2 Table in S1 File) [16]. All articles were independently assessed by two reviewers. Most studies were from moderate to high quality.

### Statistical analysis

Since the estimated results of random effects model had larger confidence intervals than those of fixed-effects models, we used a random effects model to generate odds ratio [17]. Two separate analyses were conducted: DN predicting depression and depression predicting DN. The multivariable-adjusted effect estimated OR with 95% CI was used as a common measure. Statistical heterogeneity between the studies was tested with the Cochran Q and I^2^ indices. Subgroup analyses was used to explore potential variability, with subgroups including type of diabetes, study design, age, country, method of depression assessment, evaluation of DN, classification of depression. Begg’s test was used to assess the publication bias. All analyses were calculated by using STATA 13.0 software (Stata Corporation, College Station, TX, USA), and the statistical significance was set a priori at P ≤ 0.05.

## Results

### Literature search results

Our search strategy yielded 32,108 articles, of which 9,759 were found to be duplicate records and were therefore excluded. In addition, 22,304 articles were excluded on the account that they were reviews, meta-analyses, conferences abstracts, case reports, animal experiments or irrelevant studies. Consequently, 45 articles were eligible for full-text review and data assessment. Among them, 15 were excluded due to the following reasons: 7 lacked sufficient data to generate reliable risk estimates, and 8 described the relationship between depression and diabetes/diabetes complications (Fig 1). Ultimately, 30 studies were investigated, where 24 were about DN predicting depression [18–41] and 6 were about depression predicting DN [42–47] (Tables 1 and 2). It was noted that 4 of the final 30 studies reported HR and one reported RR in risk evaluation [27]. Since OR was a commonly used indicator in case-control studies in epidemiology [48], the reported RR/HR data was regarded as OR as an approximation [49].

**Table 1:** Studies of diabetic nephropathy (DN) and incident depression.

Study	Country	Study design	DN/Total	Mean age	Depression assessment	DN assessment	Odds ratio (95% CI)
van Steenbergen-Weijenburg 2011	Netherlands	cross-sectional	194/596	63.2±12.6	PHQ-9	NR	1.75 (1.10–2.77)
Habtewold 2016	Ethiopia	cross-sectional	69/264	43.9±10.9	PHQ-9	NR	2.90 (1.20–6.70)
Takasaki 2016	Japan	cross-sectional	2,212/2,212	60.9±13.7	PHQ-9	Albuminuria+eGFR	1.20 (1.06–1.37)^d1^
							1.22 (1.03–1.46)^d2^
							0.97 (0.84–1.12)^e1^
							1.02 (0.84–1.23)^e2^
Ishizawa 2016	Japan	cross-sectional	NR/4,283	73.0 ± 6.0	PHQ-9	NR	1.79 (0.87–3.69)^f^
							5.12 (2.62–10.00)^g^
Campbell 2013	America	cross-sectional	4,583/5,805	67.2±4.8	PHQ-8	eGFR	2.04 (1.06–3.91)
W. Katon 2009	America	cohort	987/2,759	> 60(55.1%)^b^	PHQ-9	NR	1.06 (0.65–1.71)
Wang 2017	China	cross-sectional	NR/210	57.7 ± 11.7	HADS	Albuminuria+eGFR	11.76 (3.09–44.74)
Roy 2012	Bangladesh	cross-sectional	18/417	53.2 ± 7.6	PHQ-9	NR	1.00 (0.50–3.40)
Salinero-Fort 2018	Spain	cohort	400/2,955	70.2 ±10.6	Diagnostic	NR	1.08 (0.78–1.48)
Poongothai 2011	India	cohort	NR/847	49.1±12.2	PHQ-12	UAER	1.71 (0.87–3.35)
Pouwer 2010^a1^	Netherlands	cross-sectional	57/2,055	43.0±14.0	DI	NR	1.19 (0.62–2.32)
Pouwer 2010^a2^	Netherlands	cross-sectional	133/2,050	61.0±12.0	DI	NR	0.99 (0.54–1.82)
Ahola 2020	EU	cohort	219/1,046	35^c^	BDI	UAER/UACR	1.05 (1.03–1.07)
Hirai 2012	America	cohort	235/484	49.1 ± 9.25	CES-D	NR	1.26 (0.77–2.08)
D’Amato 2016	Italy	cross-sectional	48/181	60.7 ± 11.5	BDI	eGFR	2.19 (0.57–8.45)
Yoshida 2009	Japan	cross-sectional	17/129	52.7±10.5	DI	NR	0.69 (0.23–2.11)
Sharif 2019	Pakistan	cross-sectional	14/100	58.3 ± 12.4	PHQ-9	NR	4.20 (0.83–20.1)
Rajput 2016	India	case-control	NR/410	54.7±9.9	HDRS	NR	4.41 (2.78–6.99)
Raval 2010	India	cross-sectional	106/300	54.2 ± 10.0	PHQ-9	NR	1.81 (1.02–3.21)
Khan 2019	Pakistan	cross-sectional	17/142	57.0 ± 11.2	HADS	NR	0.52 (0.18–1.49)
AlBekairy 2017	Saudi Arabia	cross-sectional	34/158	67.2 ± 12.6	HADS	NR	2.56 (1.13–5.80)
Bajaj 2012	India	case-control	37/60	47.7 ± 9.8	BDI	NR	4.73 (1.44–15.46)
Aljohani 2021	Saudi Arabia	cross-sectional	33/267	57.9 ± 8.7	PHQ-9	NR	2.97 (1.01–8.77)
Albasheer 2018	Saudi Arabia	cross-sectional	14/385	47.9 ± 11.4	PHQ-9	NR	3.07 (1.01–9.36)
Bai 2017	Canada	cohort	113/323	65.5 ±8.5	GDS	UACR+eGFR	1.32 (1.00–1.72)

DN, diabetic nephropathy; PHQ, patient health questionnaire; eGFR, estimate glomerular filtration rate; UAER, urinary albumin excretion rate; UACR, urinary albumin/creatinine ratio; HADS, hospital anxiety and depression scale; BDI, beck depression inventory; CES-D, center for epidemiological studies depression scale; HDRS, hamilton depression rating scale; GDS, **g**eriatric depression scale; DI, diagnostic interview; NR, not report.

^a1^Type 1 diabetes

^a2^Type 2 diabetes

^b^ Statistical description of age was presented as ratio

^c^ Statistical description of age was presented as mean

^d1^Assessment of DN with albuminuria: mild depression

^d2^Assessment of DN with albuminuria: moderate-severe depression

^e1^Assessment of DN with eGFR: mild depression

^e2^Assessment of DN with eGFR: moderate-severe depression

^f^ Classification of depression: mild depression

^g^ Classification of depression: moderate-severe depression.

**Table 2:** Studies of depression and incident diabetic nephropathy (DN).

Study	Country	Study design	DN/Total	Mean age	Assessment depression	Assessment DN	Odds ratio (95% CI)
Yu 2014	America	cohort	NR/3,886	64.4 ±13.6^a^	PHQ-9	NR	1.08 (0.52–2.25)^a^
				59.3 ±13.4^b^			1.85 (1.02–3.33)^b^
Yu 2013	America	cross-sectional	1,778/4,082	59.7 ±13.7	PHQ-9	Albuminuria+eGFR	1.34 (1.02–1.75)^d^
							1.07 (0.80–1.43)^e^
Novak 2016	America	cohort	180,343/933,211	65.0±10.0	Diagnostic	eGFR	1.18 (1.17–1.20)
Pan 2017	China	cross-sectional	20/288	63.2± 10.5	HAM-D 24	UAER	4.89 (1.13–21.23)
Ahola 2021	Finland	cohort	688/3,730	38.6^c^	Diagnostic	UAER	1.32 (1.08–1.60)
Horiba 2022	Japan	cohort	NR/486	67.0±12.0	PHQ-9	Albuminuria+eGFR	1.12 (0.76–1.66)^a^
							1.45 (0.93–2.27)^b^

HAM-D 24, hamilton rating scale for depression-24; PHQ, patient health questionnaire; UAER, urinary albumin excretion rate; eGFR, estimate glomerular filtration rate; NR, not report.

^a^ Classification of depression: mild depression

^b^ Classification of depression: moderate-severe depression

^c^ Statistical description of age was presented as mean.

^d^ Assessment of DN with albuminuria

^e^ Assessment of DN with eGFR.

### Study characteristics and quality assessment

The features of the screened studies were gathered in Tables 1 and 2. In the 30 studies published between 2009 and 2020 were involved 974,121 participants. Six studies were from Europe [21, 23, 28, 37, 40, 46], one from Africa [29], 16 from Asia [19, 20, 22, 24, 26, 30–33, 35, 36, 38, 39, 41, 45, 47], and 7 from America [18, 25, 27, 34, 42–44]. There were 10 prospective cohort studies [18, 22, 25, 35, 37, 40, 43, 44, 46, 47], 18 cross-sectional studies [19–21, 23, 26–30, 32, 33, 35, 36, 38, 39, 41], 2 case-control studies [23, 31]. The largest number of participants were 933,211, while the longest follow-up time was 25 years. Cross-sectional, case-control, cohort studies were evaluated by AHRQ and NOS assessment, respectively. Results of quality assessments in the included studies are shown in S1-S3 Tables in S1 File. All included studies exhibited moderate to high qualities. Therefore, 30 studies were included in the meta‐analysis (Tables 1 and 2).

There was 1 study analyzed the relationship between type 1 and type 2 DN and depression with two OR values and 95% confidence interval reported, this report was divided into two groups that were pooled with other studies [21]. Similarly, for the study investigating the relationship between albuminuria/eGFR and depression, the data were divided for two groups as well [42]. According to the conditions of patients, depression was marked as mild, moderate, and severe in 3 studies, we categorized the moderate and severe depression as one group [30, 43, 47]. For the literature that analyzed the relationship between albuminuria/eGFR and the mild, moderate/severe depression, the data were divided for four groups [32]. Thus, a total of 38 groups from 30 studies were included in our meta-analysis (Tables 1 and 2).

### DN predicting depression

**Fig 2:**
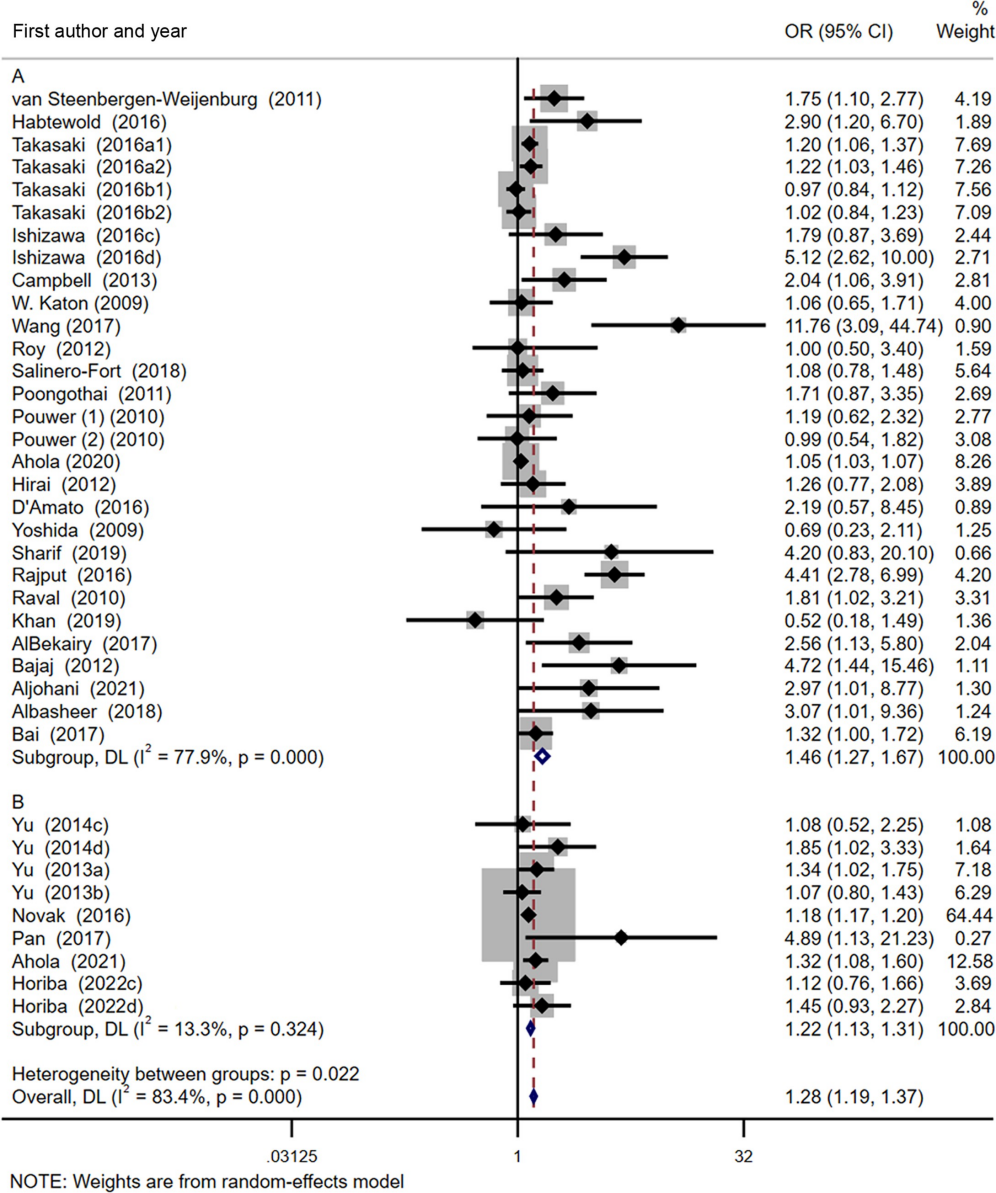
Forest plot of depression and diabetic nephropathy A: diabetic nephropathy predicting incident depression. B: depression predicting incident diabetic nephropathy.

The result of meta-analysis showed a significant link between DN and depression (Fig 2). Analysis of the 28,438 participants in 24 studies showed DN predicting depression (Fig 2A). The pooled OR was 1.46 (95% CI: 1.27–1.67), showing that DN may be an apparent risk factor for depression. However, a significant heterogeneity was identified across the included studies (P *=* 0.000, I^2^ = 77.9%) (Fig 2A).

### Depression predicting DN

In 945,683 participants of 6 studies were shown that depression predicted DN (Fig 2B). Interestingly, a relatively low heterogeneity was noted across the studies (P *=* 0.324, I^2^ = 13.3%). As indicated by the pooled OR value of 1.22 (95% CI: 1.13–1.31), depression may be an indicator for DN (Fig 2B).

### Subgroup analysis

Considering the significant heterogeneity across the studies on DN predicting depression, a series of random effects on subgroup analyses was explored in the identified association across groups. As shown in Table 3, the studies were categorized as subgroups based on the type of DN, study design, age, country, method of depression assessment, diagnostic methods of DN, and classification of depression. Subgroup analyses showed that T2DM-induced DN (OR 1.95, 95% CI: 1.43–2.66) exhibited a higher risk for depression than that induced by T1DM (OR 1.08, 95% CI: 0.99–1.19). Moreover, the case-control study (OR 1.49, 95% CI: 1.06–1.92) showed the highest risks of among different study designs. Furthermore, the DN participants from Africa were more prone to depression (OR 2.9, 95% CI: 1.23–6.85) than those from other areas, suggesting that the diagnostic and assessment methods for DN and depression showed high impacts on the risk estimates.

**Table 3:** Results of subgroup analysis about (DN) and incident depression.

Group	Number of Studies	OR	95% CI	Z	P	I^2^ (%)	P for heterogeneity
Type of DN							
T2DM induced DN	17	1.95	1.43–2.66	4.18	0.000	70.4	0.000
T1DM induced DN	4	1.08	0.99–1.19	1.72	0.085	10.9	0.338
NR	8	1.25	1.02–1.54	2.17	0.030	76.5	0.000
Study design							
Cross-sectional	21	0.41	0.23–0.60	4.36	0.000	70.8	0.000
Cohort	6	0.07	0.01–0.13	2.18	0.029	4.8	0.386
Case-control	2	1.49	1.06–1.92	6.81	0.000	0.0	0.915
Age							
≥60	14	1.30	1.12–1.51	3.40	0.001	67.8	0.000
<60	15	1.90	1.30–2.76	3.32	0.000	82.4	0.000
Country							
Europe	6	1.11	0.97–1.26	1.46	0.143	17.0	0.304
Africa	1	2.90	1.23–6.85	2.43	0.015	0.0	0.000
Asia	18	1.74	1.38–2.20	4.62	0.000	82.1	0.000
America	4	1.31	1.07–1.61	2.64	0.008	0.0	0.470
Method of depression assessment							
Self-reported questionnaire	25	1.55	1.33–1.80	5.76	0.000	81.0	0.000
Diagnosis by a physician	1	1.08	0.78–1.48	0.45	0.650	0.0	0.000
Diagnostic interview	3	1.01	0.67–1.53	0.06	0.951	0.0	0.712
Diagnostic methods of DN							
NR	19	1.77	1.33–2.36	3.89	0.000	70.4	0.000
UAER/UACR/albuminuria	4	1.15	1.02–1.29	2.26	0.024	65.7	0.033
eGFR	4	1.08	0.87–1.34	0.71	0.475	50.2	0.110
UAER/UACR/albuminuria/eGFR	2	3.56	0.42–30.04	1.17	0.244	89.9	0.002
Classification of depression							
NR	23	1.65	1.32–2.05	4.44	0.000	77.1	0.000
Mild	3	1.12	0.91–1.39	1.07	0.283	69.0	0.040
Moderate-severe	3	1.58	0.98–2.56	1.86	0.063	90.4	0.000

DN, diabetic nephropathy; T2DM, type 2 diabetes; T1DM, type 1 diabetes; NR, not report; UAER, urinary albumin excretion rate; UACR, urinary albumin/creatinine ratio; eGFR: estimate glomerular filtration rate.

### Publication bias

**Fig 3:**
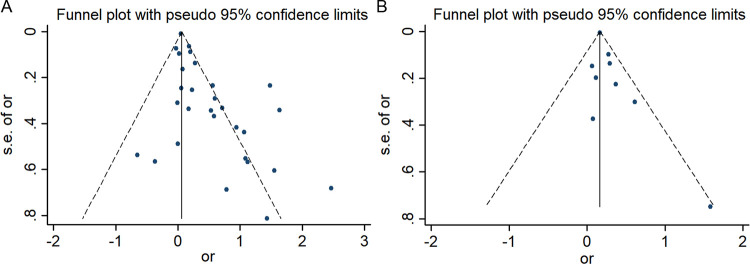
Funnel plot: A: diabetic nephropathy predicting incident depression. B: depression predicting incident diabetic nephropathy.

**Fig 4:**
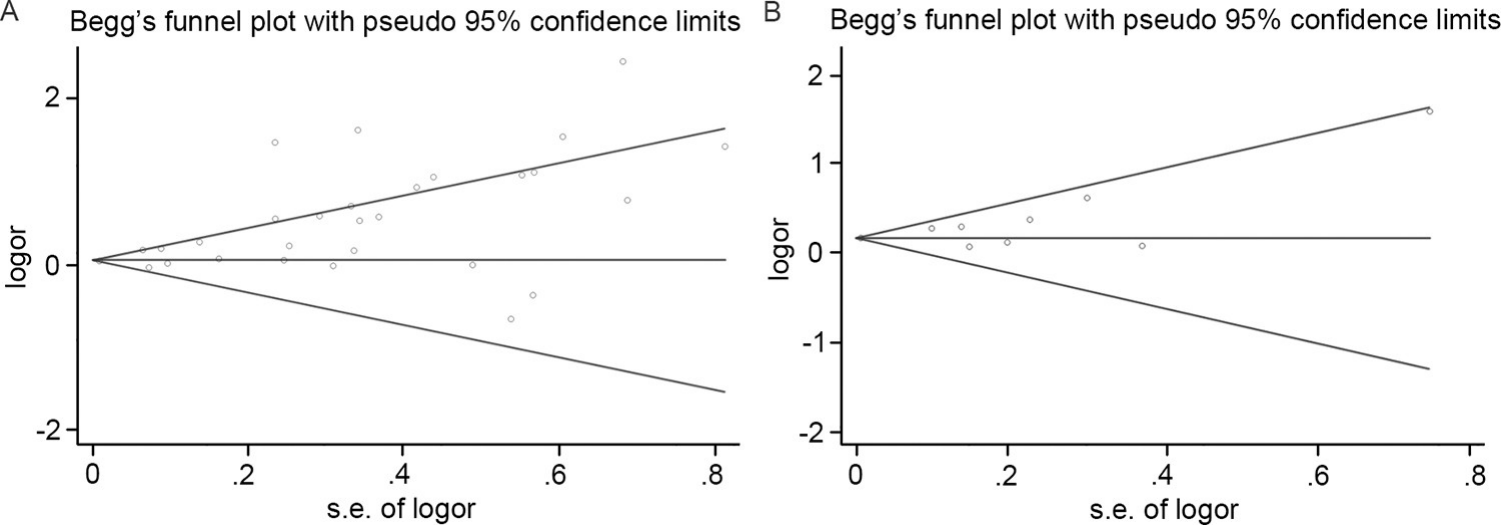
Begg’s funnel plot with 95% confidence limits. A: diabetic nephropathy predicting incident depression. B: depression predicting incident diabetic nephropathy.

The publication bias of the included studies was evaluated using the funnel plot (Fig 3). Additionally, the Begg’s test was used to measure its potential symmetry (Fig 4). The funnel plot and statistical analysis showed an apparent publication bias in DN predicting depression (Begg’s test, P = 0.038, Figs 3A and 4A). However, for the analysis of depression predicting DN, the funnel plot together with the Begg’s test (P = 0.175) did not indicate any publication bias of the included studies (Figs 3B and 4B).

## Discussion

Previous studies show associations between depression and diabetes, with type 2 diabetes increasing a 24% risk of developing depression [14], and depression increasing a 60% risk of developing type 2 diabetes [12]. Through a systematic review and meta-analysis, the present study investigated a bidirectional association between depression and diabetes. Our meta-analysis shows that DN may be a significant risk factor of depression. When considering the different evaluation methods for DN, studies using the UAER/UACR/albuminuria as assessment criteria showed promoted increase in the risk estimate compared to those assessed by eGFR (Table 3). The result was in agreement with Yu’s cohort study, where a higher prevalence of microalbuminuria instead of eGFR was found in relation to depression [42]. Albuminuria was a risk factor of cardio-cerebrovascular disease [50], and cardio-cerebrovascular disease could aggravate the condition of patients with advanced kidney disease, leading to depression [51]. Hedayati et al. found a close relationship between depression and eGFR. Veterans with chronic kidney disease had a 21% increased risk of major depression in the United States [52]. This difference may be related to different study populations and study designs. The mechanisms underlying the comorbidity of DN and depression remain unclear. What specific biological or psychological factors that would mediate a specific, and bidirectional association between DN and depression is still intriguing. Similar to the established link between diabetes mellitus and depression, the mechanism between DN and depression may potentially involve factors of serious complication of diabetes and effect of patient therapies such as dialysis or kidney transplantation [53]. While patients with kidney failure experienced breathing difficulties, fatigue, or poor appetite, which rendered patients more prone to depression [27, 54], the risk of depression appeared reduced after a successful kidney transplant [55].

Depression impacts every aspect of human lives including social, psychological, behavioral, and biological activities. Our present study suggests an overall mild association between depression and the incidence of DN (Fig 2). Yu MK’s et al. reported that major depression led to an 85% increased risk of ESRD in patients with type 2 diabetes, while mild depression had no increased risk [43]. Hedayati et al. found that for the male veterans with CKD, major depression led to a 3.5-fold increased risk of chronic dialysis [52]. Tsai et al. showed that severe depressive symptoms in patients with CKD leaded to a faster decline in renal function, ESRD, or death [53]. All these reports support the model of depression coexisting as a comorbidity of DN. In addition, studies have revealed that depression-related behaviors and medications for depression lead to DN [56]. Similar to depression with diabetes mellitus, the mechanism of DN pathogenesis may be related to the biological and behavioral risk factors caused by depression [57]. Once depression occurs in diabetic patients, their self-care abilities such as diet, exercise, blood glucose monitoring, and so on will decline, leading to the development of DN [58–61]. Besides, depression relates to the disorder of immune system, and the expression of inflammation markers (IL-6, C-reactive protein, TNF-α) increases the risk of DN [62–66]. Furthermore, depression increases the activities of the hypothalamic-pituitary-adrenal axis, sympathetic nervous system, the stress hormone, cortisol excites glucose production, fat decomposition, free fatty acids circulation, and reduces insulin secretion and sensitivity, potentially resulting increased risk of DN [67–69].

### Limitation of the study

The present study suggests for the first time a bidirectional risk development between DN and depression by meta-analysis of meaningful studies and participants. The findings are generally in agreement with the reported cases of illness and of value for consideration in the management of DN and depression. However, there are several limitations in this study for cautious interpretation. First, the quality of related studies is of limitation, with potential heterogeneity and publication bias. Although we chose adjusted estimates to analyze the DN-depression relationship, confounders may be adjusted differently by different investigators, leading to varied statistic result. Variability in adjustment for confounding factors, could be a possible source of heterogeneity. Second, DN and depression might be assessed differently in different studies that potentially influenced the result. For the diagnostic methods of DN, 18 studies did not describe any specific evaluation method [18–21, 23–26, 29–31, 33, 36–39, 41, 43], 5 studies used UAER/UACR/albuminuria to assess renal function [22, 33, 40, 45, 46], and 4 studies used eGFR [27, 28, 32, 44]. For the depression assessment, diagnostic interview was a gold standard for major depression, only 2 studies adopted this method [19, 21]. Additionally, 25 studies used different cutoff scores to assess depression [18, 20, 22–36, 38–43, 45, 47], some cutoff scores were not commonly used. Moreover, depression might change over time, and dynamic scores were unavailable. Three studies accessed depression by general practitioner’s diagnosis. However, as reported, approximately 50% of depressive symptoms were overlooked in general practice [37, 44, 45]. Furthermore, patients with depression tended to go to the hospital more frequently and diagnostic bias could not be excluded with impact on the results.

## Conclusion

This meta-analysis shows that the relationship between DN and depression may be bidirectional. DN may be a sensitive risk factor of depression, and depression may be also a predictor of DN. The mechanisms underlying this relationship warrant further investigation, and so is clinical study in the comorbidity of depression and DN.

## Supporting information

S1 Checklist PRISMA 2020 checklist. (DOCX)

S1 File Quality assessment. (DOCX)

S2 File Search strategy. (DOC)

S1 Fig PRISMA flow chart. (TIF)

S1 Data (XLS)
