# Supplementary material for: Bidirectional association between depression and diabetic nephropathy by meta-analysis
Source: PLoS One. 2022 Dec 20;17(12):e0278489. doi: 10.1371/journal.pone.0278489 (PMC9767359; doi:10.1371/journal.pone.0278489)
Supplement: S1 File — (DOCX) [file pone.0278489.s002.docx]

**Bidirectional association between depression and diabetic nephropathy by meta-analysis**

Tingting Fang^1,2*^, Qiuling Zhang^3^, Zhiguo Wang^1^, Jun-Ping Liu^1,4,5*^

^1^Institute of Ageing Research, Hangzhou Normal University, School of Basic Medicine, Hangzhou, Zhejiang Province 311121, China

^2^School of Public Health, Han gzhou Normal University, Hangzhou, Zhejiang Province 311121, China

^3^Department of Endocrinology, the Affiliated Hospital of Hangzhou Normal University, Hangzhou, Zhejiang Province 311121, China

^4^Monash University Department of Immunology and Pathology, Central Clinical School, Faculty of Medicine, Commercial Road, Prahran, Victoria 3018, Australia

^5^Hudson Institute of Medical Research, Clayton, Victoria 3168, Australia

^*^ Corresponding authors:

Tingting Fang, 2020011012011@stu.hznu.edu.cn

Jun-Ping Liu, [jun-ping.liu@hznu.edu.cn](mailto:jun-ping.liu@hznu.edu.cn). Orcid number: 0000-0001-7442-2116

Short title: Bidirectional association between depression and diabetic nephropathy

**Table S1.** AHRQ assessment for cross-sectional studies.

| Author and year | Q1 | Q2 | Q3 | Q4 | Q5 | Q6 | Q7 | Q8 | Q9 | Q10 | Q11 | Quality/Score |
| --- | --- | --- | --- | --- | --- | --- | --- | --- | --- | --- | --- | --- |
| van Steenbergen-Weijenburg 2011 | 1 | 1 | 1 | 0 | 0 | 2 | 1 | 1 | 0 | 0 | 0 | Medium（7 points) |
| Habtewold 2016 | 1 | 1 | 1 | 0 | 0 | 0 | 1 | 1 | 0 | 0 | 0 | Medium（5 points) |
| Takasaki 2016 | 1 | 1 | 1 | 0 | 0 | 2 | 1 | 1 | 0 | 0 | 0 | Medium（7 points) |
| Ishizawa 2016 | 1 | 1 | 1 | 0 | 0 | 2 | 0 | 1 | 0 | 0 | 0 | Medium（6 points) |
| Campbell 2014 | 1 | 0 | 1 | 0 | 0 | 1 | 1 | 1 | 0 | 0 | 0 | Medium（5 points) |
| Wang 2017 | 1 | 1 | 1 | 0 | 0 | 1 | 0 | 1 | 0 | 0 | 0 | Medium（5 points) |
| Roy 2012 | 1 | 1 | 1 | 0 | 0 | 0 | 0 | 1 | 0 | 0 | 0 | Medium（4 points) |
| D'Amato 2016 | 1 | 1 | 1 | 0 | 0 | 1 | 0 | 1 | 0 | 0 | 0 | Medium（5 points) |
| Yoshida 2009 | 1 | 1 | 1 | 0 | 0 | 1 | 0 | 1 | 0 | 0 | 0 | Medium（5 points) |
| Sharif 2019 | 1 | 1 | 1 | 0 | 0 | 1 | 0 | 1 | 0 | 0 | 0 | Medium（5 points) |
| Raval 2010 | 1 | 1 | 1 | 0 | 0 | 1 | 0 | 1 | 0 | 0 | 0 | Medium（5 points) |
| Khan 2019 | 1 | 1 | 1 | 0 | 0 | 1 | 0 | 1 | 0 | 0 | 0 | Medium（5 points) |
| AlBekairy 2017 | 1 | 1 | 1 | 0 | 0 | 1 | 0 | 1 | 0 | 0 | 0 | Medium（5 points) |
| Aljohani 2021 | 1 | 1 | 1 | 0 | 0 | 1 | 0 | 0 | 0 | 0 | 0 | Medium（4 points) |
| Albasheer 2018 | 1 | 1 | 1 | 0 | 0 | 1 | 0 | 0 | 0 | 0 | 0 | Medium（4 points) |
| Yu 2013 | 1 | 1 | 1 | 0 | 0 | 1 | 1 | 1 | 0 | 1 | 0 | Medium（7 points) |
| Pan 2017 | 1 | 1 | 1 | 0 | 0 | 1 | 0 | 1 | 0 | 0 | 0 | Medium（5 points) |
| Pouwer 2010 | 1 | 1 | 1 | 0 | 0 | 1 | 1 | 1 | 0 | 1 | 0 | Medium（7 points) |

Notes: Q1. Define the source of information (survey, record review); Q2. List inclusion and exclusion criteria for exposed and unexposed subjects (cases and controls) or refer to previous publications; Q3. Indicate time period used for identifying patients; Q4. Indicate whether or not subjects were consecutive if not population-based; Q5. Indicate if evaluators of subjective components of study were masked to other aspects of the status of the participants; Q6. Describe any assessments undertaken for quality assurance purposes (e.g., test/retest of primary outcome measurements); Q7. Explain any patient exclusions from analysis; Q8. Describe how confounding was assessed and/or controlled; Q9. If applicable, explain how missing data were handled in the analysis; Q10. Summarize patient response rates and completeness of data collection; Q11. Clarify what follow-up, if any, was expected and the percentage of patients for which incomplete data or follow-up was obtained.

**Table S2.** NOS assessment for cohort Studies.

| Author(year) | Selection | | | | Comparability | Exposure | | | Quality/ score |
| --- | --- | --- | --- | --- | --- | --- | --- | --- | --- |
|  | Q1 | Q2 | Q3 | Q4 | Q5 | Q6 | Q7 | Q8 |  |
| W. Katon 2009 | 1 | 1 | 1 | 1 | 2 | 1 | 1 | 1 | High（9 points） |
| Salinero-Fort 2018 | 1 | 1 | 1 | 1 | 2 | 1 |  |  | High（7 points） |
| Ahola 2020 | 1 | 1 | 1 | 1 | 2 | 1 |  |  | High（7 points） |
| Hirai 2012 | 1 | 1 | 1 | 1 | 2 | 1 | 1 | 1 | High（9 points） |
| Bai 2017 | 1 | 1 | 1 | 1 | 2 | 1 |  |  | High（7 points） |
| Yu 2014 | 1 | 1 | 1 | 1 | 2 | 1 | 1 | 1 | High（9 points） |
| Novak 2016 | 1 | 1 | 1 | 1 | 2 | 1 | 1 | 1 | High（9 points） |
| Ahola 2021 | 1 | 1 | 1 | 1 | 2 | 1 | 1 | 1 | High（9 points） |
| Horiba 2021 | 1 | 1 | 1 | 1 | 2 | 1 | 1 | 1 | High（9 points） |

Notes: Q1.The representative of the exposure group (one point); Q2. Selection methods for non-exposed groups (one point); Q3. Methods for determining exposure factors (one point); Q4. Identify no outcome indicators to be observed at the start of the study (one point); Q5. Comparability between exposed and unexposed groups was considered in the design and statistical analysis (two points); Q6. The adequacy of the evaluation of the results (one point); Q7. Whether follow-up was long enough after the outcome occurred (one point); Q8. Adequacy of follow-up in exposed and non-exposed groups (one point).

**Table S3.** NOS assessment for case-control studies.

| **Author(year)** | Selection | | | | Comparability | Exposure | | | **Quality/ score** |
| --- | --- | --- | --- | --- | --- | --- | --- | --- | --- |
|  | Q1 | Q2 | Q3 | Q4 | Q5 | Q6 | Q7 | Q8 |  |
| Rajput 2016 | 1 | 1 | 1 | 0 | 2 | 1 | 0 | 0 | Medium ( 6 points） |
| Bajaj 2012 | 1 | 1 | 1 | 1 | 2 | 1 | 1 | 0 | High ( 8 points) |

Notes: Q1. Whether case determination is appropriate; Q2. Representativeness of cases; Q3. Control selection; Q4. Determination of control; Q5. Consider the comparability of cases and controls when designing and making a statistical analysis; Q6. Determination of the exposure factors; Q7. The same methods were used to determine the exposure factors in the cases and controls; Q8. No response rate.
