# Supplementary material for: Bidirectional association between depression and diabetic nephropathy by meta-analysis
Source: PLoS One. 2022 Dec 20;17(12):e0278489. doi: 10.1371/journal.pone.0278489 (PMC9767359; doi:10.1371/journal.pone.0278489)
Supplement: S2 File — (DOC) [file pone.0278489.s003.doc]

**Bidirectional association between depression and diabetic nephropathy by meta-analysis**

Tingting Fang^1,2*^, Qiuling Zhang^3^, Zhiguo Wang^1^, Jun-Ping Liu^1,4,5*^

^1^Institute of Ageing Research, Hangzhou Normal University, School of Basic Medicine, Hangzhou, Zhejiang Province 311121, China

^2^School of Public Health, Han gzhou Normal University, Hangzhou, Zhejiang Province 311121, China

^3^Department of Endocrinology, the Affiliated Hospital of Hangzhou Normal University, Hangzhou, Zhejiang Province 311121, China

^4^Monash University Department of Immunology and Pathology, Central Clinical School, Faculty of Medicine, Commercial Road, Prahran, Victoria 3018, Australia

^5^Hudson Institute of Medical Research, Clayton, Victoria 3168, Australia

^*^ Corresponding authors:

Tingting Fang, 2020011012011@stu.hznu.edu.cn

Jun-Ping Liu, [jun-ping.liu@hznu.edu.cn](mailto:jun-ping.liu@hznu.edu.cn). Orcid number: 0000-0001-7442-2116

Short title: Bidirectional association between depression and diabetic nephropathy

S4 Search strategy

Search Terms in PubMed

| Search | Results |
| --- | --- |
| #1 ("Diabetic Nephropathies"[Mesh]) OR (((((((((((((((((Nephropathies, Diabetic[Title/Abstract]) OR (Nephropathy, Diabetic[Title/Abstract])) OR (Diabetic Nephropathy[Title/Abstract])) OR (Diabetic Kidney Disease[Title/Abstract])) OR (Diabetic Kidney Diseases[Title/Abstract])) OR (Kidney Disease, Diabetic[Title/Abstract])) OR (Kidney Diseases, Diabetic[Title/Abstract])) OR (Diabetic Glomerulosclerosis[Title/Abstract])) OR (Glomerulosclerosis, Diabetic[Title/Abstract])) OR (Intracapillary Glomerulosclerosis[Title/Abstract])) OR (Nodular Glomerulosclerosis[Title/Abstract])) OR (Glomerulosclerosis, Nodular[Title/Abstract])) OR (Kimmelstiel-Wilson Syndrome[Title/Abstract])) OR (Kimmelstiel Wilson Syndrome[Title/Abstract])) OR (Syndrome, Kimmelstiel-Wilson[Title/Abstract])) OR (Kimmelstiel-Wilson Disease[Title/Abstract])) OR (Kimmelstiel Wilson Disease[Title/Abstract]) OR (DN[Title/Abstract]) OR (DKD[Title/Abstract]) OR (Nephropathy[Title/Abstract]) OR (Nephrosis[Title/Abstract]) OR (Chronic Kidney Disease[Title/Abstract]) OR (CKD[Title/Abstract])) | 153,360 |
| #2 ((((((((((((Diabetes Mellitus[Title/Abstract]) OR (Diabetes[Title/Abstract])) OR (Diabetes Mellitus, Type 2[Title/Abstract])) OR (Diabetes Mellitus, Type 1[Title/Abstract])) OR (Diabetes Complication[Title/Abstract])) OR (Diabetes-Related Complications[Title/Abstract])) OR (Diabetes Related Complications[Title/Abstract])) OR (Diabetes-Related Complication[Title/Abstract])) OR (Diabetic Complications[Title/Abstract])) OR (Diabetic Complication[Title/Abstract])) OR (Complications of Diabetes Mellitus[Title/Abstract])) OR (Diabetes Mellitus Complication[Title/Abstract])) OR (Diabetes Mellitus Complications[Title/Abstract]) | 625,821 |
| #3 (((((((((((((((((((((((((((("Depression"[Mesh]) OR (Depressive Symptoms[Title/Abstract])) OR (Depressive Symptom[Title/Abstract])) OR (Symptom, Depressive[Title/Abstract])) OR (Symptoms, Depressive[Title/Abstract])) OR (Emotional Depression[Title/Abstract])) OR (Depression, Emotional[Title/Abstract])) OR (Tristimania[Title/Abstract])) OR (Depressive Disorders[Title/Abstract])) OR (Disorder, Depressive[Title/Abstract])) OR (Disorders, Depressive[Title/Abstract])) OR (Neurosis, Depressive[Title/Abstract])) OR (Depressive Neuroses[Title/Abstract])) OR (Depressive Neurosis[Title/Abstract])) OR (Neuroses, Depressive[Title/Abstract])) OR (Depression, Endogenous[Title/Abstract])) OR (Depressions, Endogenous[Title/Abstract])) OR (Endogenous Depression[Title/Abstract])) OR (Endogenous Depressions[Title/Abstract])) OR (Depression, Neurotic[Title/Abstract])) OR (Depressions, Neurotic[Title/Abstract])) OR (Neurotic Depression[Title/Abstract])) OR (Neurotic Depressions[Title/Abstract])) OR (Melancholia[Title/Abstract])) OR (Melancholias[Title/Abstract])) OR (Unipolar Depression[Title/Abstract])) OR (Depression, Unipolar[Title/Abstract])) OR (Depressions, Unipolar[Title/Abstract])) OR (Unipolar Depressions[Title/Abstract]) | 190,086 |
| #4 (#1 or #2) AND #3 | 6,191 |
