# Supplementary figures and images for: Bidirectional association between depression and diabetic nephropathy by meta-analysis
Source: PLoS One. 2022 Dec 20;17(12):e0278489. doi: 10.1371/journal.pone.0278489 (PMC9767359; doi:10.1371/journal.pone.0278489)

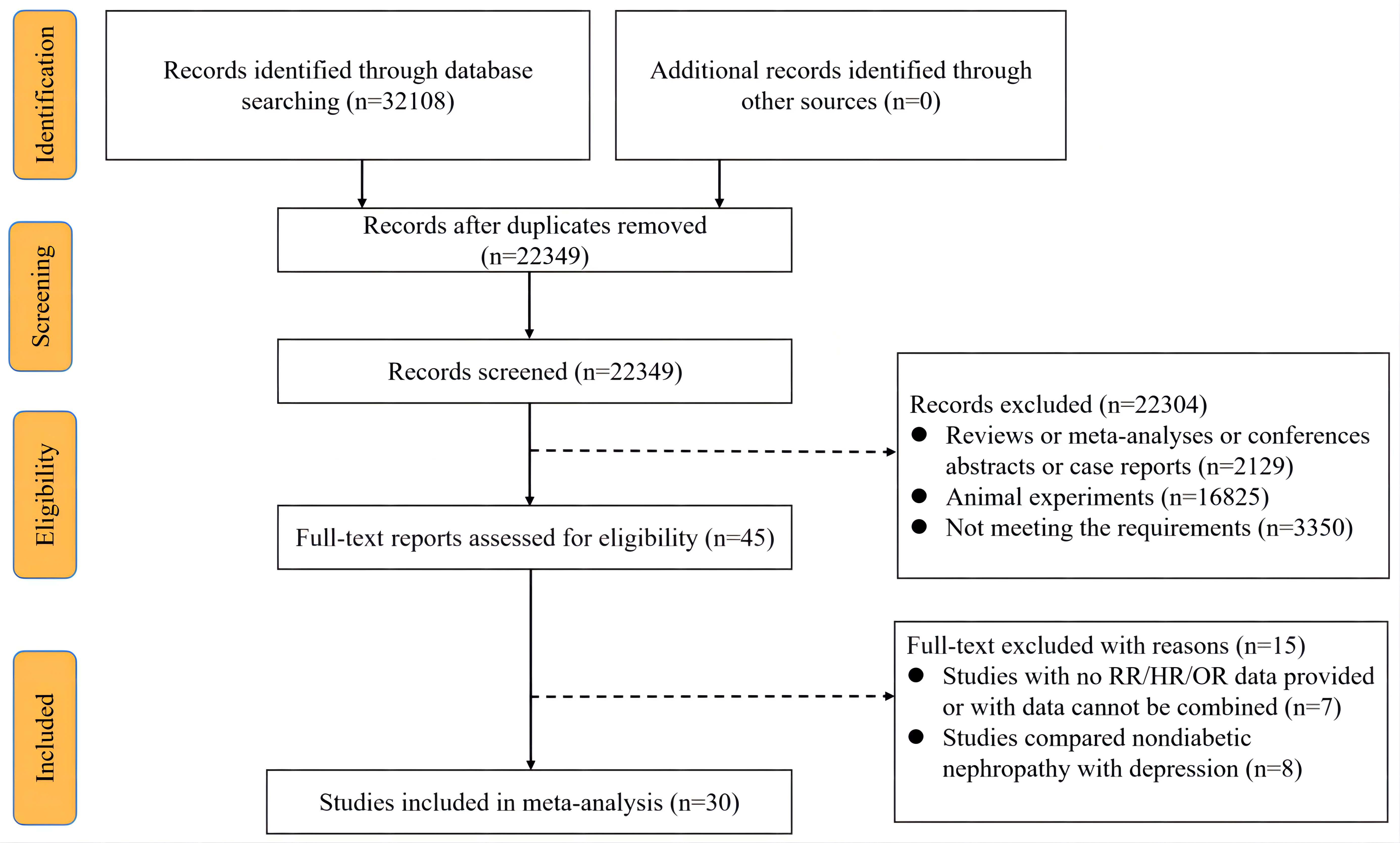

Supplement: S1 Fig — (TIF) [file pone.0278489.s004.tif]
